# Cranio-orbital craniotomy via an eyebrow incision for resection of anterior clinoid and tuberculum sella meningiomas

**DOI:** 10.1007/s00701-026-06997-y

**Published:** 2026-08-11

**Authors:** Robert A. Riestenberg, Jonathan B. Lamano, Keira Lightbody, Dennis Lee, Jared Clouse, Aden P. Haskell-Mendoza, Orin Bloch

**Affiliations:** 1 Department of Neurological Surgery, University of California, Davis, 4860 Y Street, Suite 3740, Sacramento, CA 95817 USA; 2 Department of Neurological Surgery, Northwestern University, Evanston, IL USA; 3 Department of Neurological Surgery, Stanford University, Stanford, CA USA; 4 Geisinger Health System, Department of Neurological Surgery, Danville, PA USA; 5 Department of Neurological Surgery, Duke University, Durham, NC USA

**Keywords:** Meningioma, Tuberculum sella, Anterior clinoid, Visual outcomes, Minimally invasive, Fronto-orbital, Eyebrow craniotomy

## Abstract

**Background:**

Anterior clinoid meningiomas (ACM) and tuberculum sella meningiomas (TSM) are anterior skull base tumors that commonly cause visual deterioration. The supraorbital keyhole approach may be used to resect these tumors but is limited by restricted intraoperative maneuverability.

**Objective:**

To describe and demonstrate the safety and efficacy of our modified minimally invasive cranio-orbital craniotomy compared to traditional approaches.

**Methods:**

All patients with an ACM or TSM treated with a modified cranio-orbital or traditional fronto-orbital (FO) craniotomy by the senior author were included. A literature review was conducted for ACMs and TSMs treated by transcranial (TC), endoscopic endonasal approach (EEA), and supraorbital keyhole (SoK) approaches. Visual outcomes, extent of resection, and complications were compared.

**Results:**

Six patients who underwent modified cranio-orbital craniotomy and 4 patients who underwent FO were included. Amongst patients with preoperative visual deficits, 2/4 modified cranio-orbital patients experienced postoperative visual improvement, compared to 3/3 FO patients (*p* = 0.43). Extent of resection was similar between groups. Complications related to superficial tissue dissection were less common in the modified cranio-orbital group (*p* = 0.03). In the literature, EEA approaches were associated with higher rates of visual improvement compared to TC or SoK approaches, but EEA was associated with higher rates of postoperative CSF leak. Rate of visual improvement in our modified cranio-orbital patients were similar to those reported in the literature.

**Conclusion:**

Our modified cranio-orbital craniotomy is a safe and effective approach that should be considered when planning for resection of ACMs and TSMs.

**Supplementary Information:**

The online version contains supplementary material available at https://doi.org/10.1007/s00701-026-06997-y.

## Introduction

Anterior clinoid meningiomas (ACMs) and tuberculum sella meningiomas (TSMs) are tumors of the anterior skull base which commonly present with visual impairment [24]. The primary goal of surgery for ACMs and TSMs is restoration or preservation of vision. Currently, most ACMs and TSMs are treated transcranially with a pterional or orbitozygomatic (OZ) approach, or endoscopically with transsphenoidal approaches [14, 31].

The supraorbital keyhole (SoK) approach through an eyebrow incision has become a popular method to access ACMs and TSMs [20, 25]. The SoK approach involves a small craniotomy above the superior orbital rim. The minimal dissection results in excellent cosmetic outcomes, low rates of retraction-related complications, and decreased patient discomfort [1, 2, 8, 13, 16, 18, 23, 35, 37]. However, this approach creates the additional challenge of dissecting the tumor through a narrow surgical corridor [20, 25, 26].

Here, we describe a modification to the SoK where the orbital rim and roof are removed with the usual supraorbital bone flap to expand the operative corridor. This approach increases access to the optic canal and anterior clinoid process for increased bony removal and optic nerve decompression. We report a single surgeon case series with this modified cranio-orbital approach for resection of ACMs and TSMs. We demonstrate its safety and efficacy in comparison to traditional open fronto-orbital craniotomy (FO) performed by the same surgeon, and to transcranial (TC), endoscopic endonasal approach (EEA), and standard SoK approaches for ACMs and TSMs in the literature via a systematic review.

## Methods

### Case series and chart review

This study was approved by the Northwestern University Institutional Review Board. Due to the retrospective nature of this study and minimal risk to the subjects, consent to participate was waived. Adult patients with ACMs or TSMs who underwent tumor resection via a modified cranio-orbital approach or traditional FO craniotomy by the senior author from 8/1/2013 through 3/31/2019 at Northwestern Memorial Hospital were included in this study. Clinical data was retrospectively extracted from the electronic record. Visual outcomes were categorized as improved, worsened, stable, or intact based on visual acuity and Humphrey visual fields at last follow up compared to pre-surgical evaluation. Stable refers to a preoperative visual deficit that remained unchanged after surgery. Intact refers to no preoperative visual deficit that remained unchanged after surgery. Extent of resection was based on intraoperative examination and immediate postoperative magnetic resonance imaging (MRI). Simpson grade I and II were considered gross total resection (GTR).

### Literature search and review

**Fig. 1:**
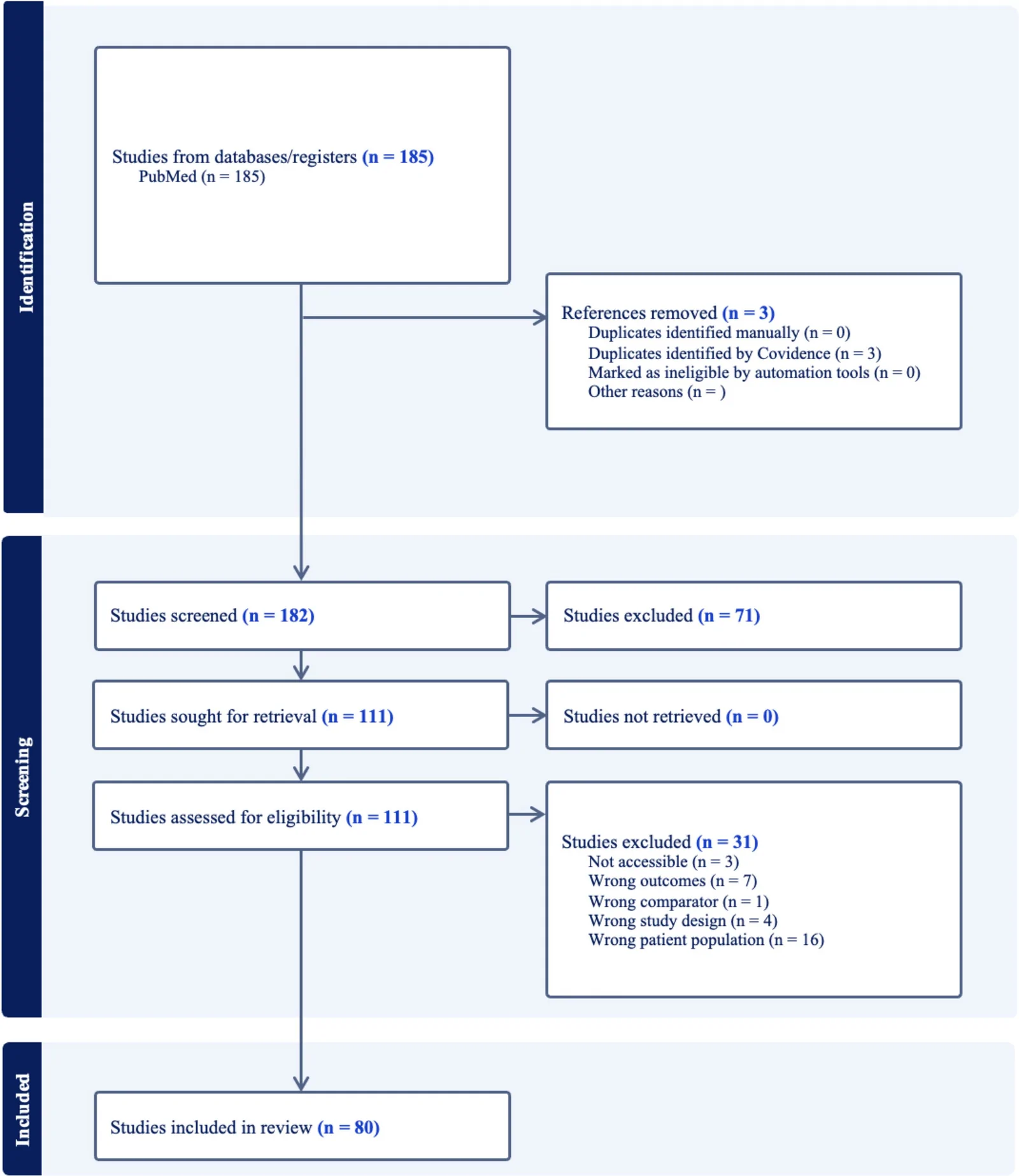
Flow diagram of studies included in systematic literature review

Pubmed was queried using the following search terms: tuberculum sellae OR tuberculum sella AND meningioma AND visual AND outcome (1/23/2025); supraorbital keyhole AND meningioma (1/23/2025); anterior clinoid AND meningioma AND visual AND outcome (1/23/2025). These terms initially yielded 185 citations. Case reports, review articles, duplicates, studies not in English, and studies published before 2009 were excluded. Articles were excluded if data for ACMs or TSMs could not be differentiated from data for other lesions, or if there was no data on visual outcomes. The analysis included 80 studies as outlined in Fig. 1. Data analogous to the case series and complications were extracted from each study. Studies were sorted into groups based on approach: TC, EEA, or SoK.

### Statistical analysis

Data management and statistical analysis in this study was accomplished using Microsoft Excel for Mac (Microsoft, Redmond, WA, USA), Prism 10 for MacOS (GraphPad, San Diego, CA, USA), and Covidence systematic review software. Mann–Whitney U tests were used to compare continuous variables, and Fisher exact tests and χ^2^ tests were used for categorical variables. Comparisons were considered statistically significant for *p* ≤ 0.05. Given the small sample size of patients included in the case series, statistical analysis of these patients were considered exploratory in nature.

## Surgical technique

**Fig. 2:**
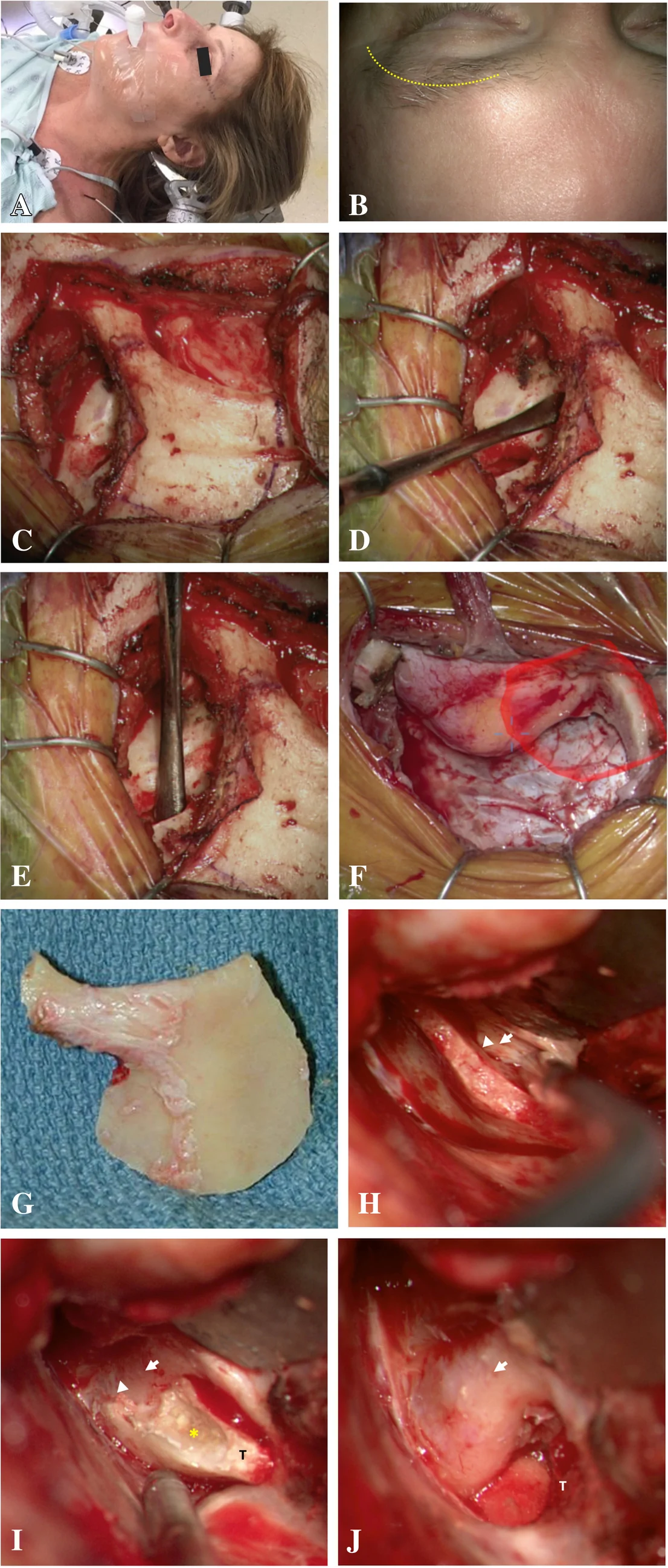
Surgical technique for modified cranio-orbital craniotomy. **A**-**J** See Surgical Technique section of text for extended description. **H**-**J** Arrow identifies optic nerve, arrowhead identifies optic canal, T denotes residual tumor. **I** Asterisk marks ACP infiltrated with tumor. **J** Decompressed optic nerve is visualized following ACP resection

The modified cranio-orbital craniotomy is performed under general anesthesia with the patient’s head affixed in Mayfield pins. The patient is positioned supine with the neck in slight extension and 15 degrees of rotation to the side contralateral to the planned craniotomy (Fig. 2A). The incision is made in the superior aspect of the brow, just lateral to the supraorbital notch, extending around the orbital rim to the lateral canthus (Fig. 2B). The frontal scalp is dissected from the pericranium and retracted superiorly. The pericranium is cut at the superior aspect of the exposure and dissected down over the orbital rim, separating the periorbita from the superior and lateral orbital walls. The temporalis muscle is incised along the superior temporal line and parallel to the lateral orbital rim, and the temporal flap is reflected laterally and inferiorly to expose the frontotemporal junction (Fig. 2C). A McCarty burr hole is performed, providing access to the intradural and intraorbital space (Fig. 2D-E). A craniotome is used to cut the bone flap from the burr hole superiorly, then medially across the frontal bone curving down to the orbital rim adjacent to and in line with the supraorbital nerve. Linear osteotomies are then made in the lateral orbital rim, connecting to the orbital portion of the McCarty burr hole, and along the superior orbital rim, connecting to the frontal craniotomy line. We utilize the bone cutting attachment on a Sonopet ultrasonic aspirator for these osteotomies to create a thin cut and reduce the risk of damaging the dura or supraorbital nerve. The superior orbital roof is fractured with an osteotome from lateral to medial through the burr hole. The supraorbital frontal bone and orbital rim are then removed as a single bone flap (Fig. 2F), uncovering the periorbital and dura (Fig. 2G). The bone flap typically includes a 3.5–4 × 2.5–3 cm piece of frontal bone above the orbital rim. The frontal dura and periorbita are further dissected off the orbital roof to allow for the piecemeal removal of the orbital roof extending to the anterior clinoid process (ACP) with Leksell rongeurs. Care is taken to avoid violation of the periorbita, thereby preventing post-operative swelling and delayed enophthalmos. Once the orbital roof is removed, the operating microscope is brought in to identify the extra-dural entrance and exit of the optic canal (Fig. 2H). The optic canal is unroofed using a high-speed self-irrigating drill with a 2-mm diamond bit. Next, the ACP is released from the skull base by continuing drilling past the lateral aspect of the optic canal down into the optic strut (Fig. 2I). When the tumor involves the dura overlying the ACP, the ACP and overlying dura are often hypervascularized, and bipolar electrocautery should be used to attain hemostasis and limit the vascular supply to the tumor. The ACP can then be removed en bloc extra-durally, creating working room around the supra-clinoid carotid and entrance to the optic canal (Fig. 2J). If the ACP and/or strut are aerated, the connection to the sinus is sealed using a muscle plug obtained from the temporalis and fibrin glue.

Once the extra-dural bony resection is complete, the dura is then opened in a U-shaped incision subfrontally and reflected inferiorly over the orbital contents. The removal of the orbital roof and ACP provide a large subfrontal working space without significant frontal lobe retraction. The tumor can then be safely dissected away from surrounding neurovascular structures and removed in a piecemeal fashion.

**Fig. 3:**
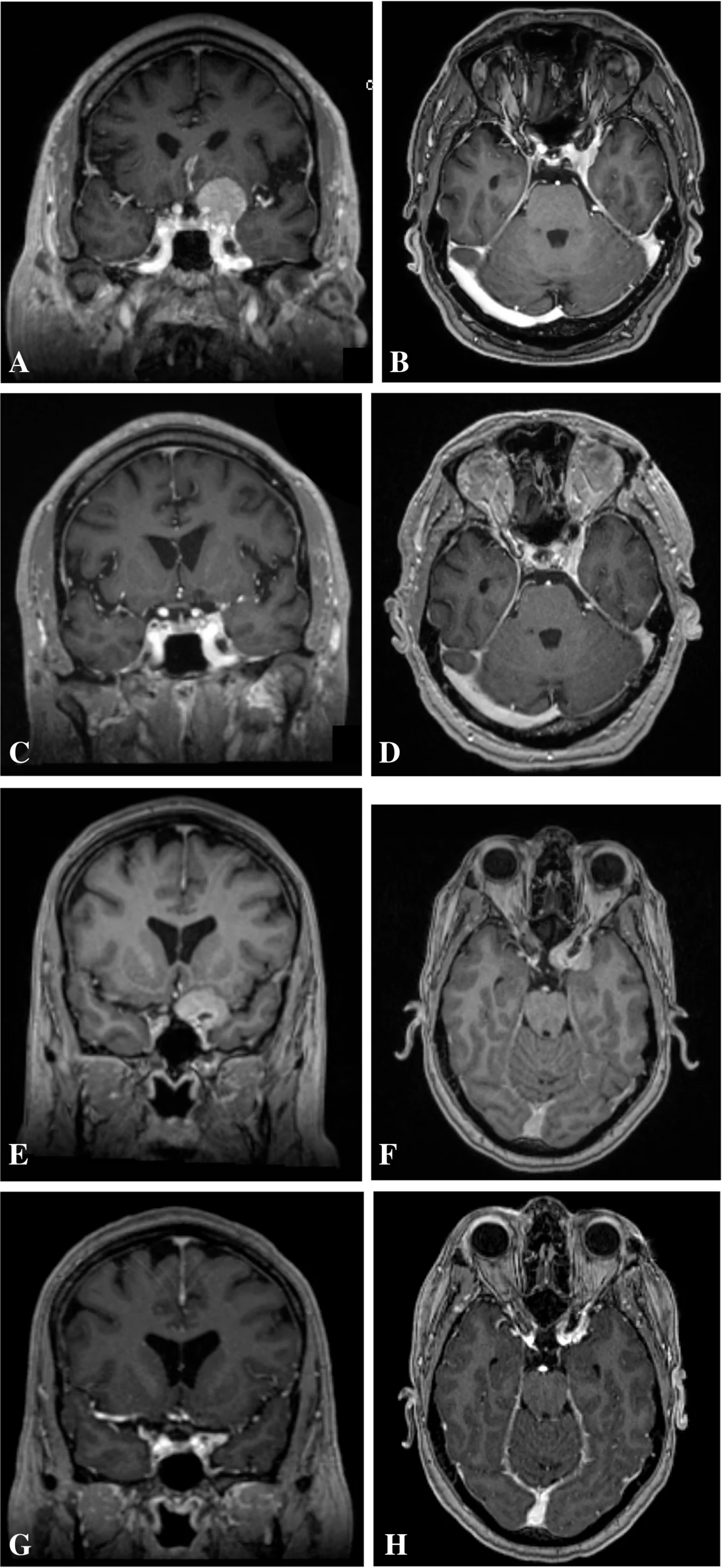
Illustrative cases of anterior clinoid meningiomas resected via modified cranio-orbital approach. Pre-operative (**A**,** B**) and post-operative (**C**,** D**) T1-weighted post-contrast images of patient 2 treated with modified cranio-orbital craniotomy for left anterior clinoid meningioma after presenting with visual changes. Residual tumor remains in the left cavernous sinus after resection. Pre-operative (**E**,** F**) and post-operative (**G**,** H**) T1-weighted post-contrast images of patient 6 treated with modified cranio-orbital craniotomy for an incidentally identified left anterior clinoid meningioma. Residual tumor remains along the dura of the anterior clinoid process extending into the optic canal

After resection of the tumor, the U-shaped dural flap is closed primarily with a 4–0 Neurolon suture. The dural closure is reinforced with on-lay Duragen and fibrin glue. The orbitofrontal bone flap is returned to its original position and fixed with titanium screws and plates. The gap around the edges of the bone flap is filled with a hydroxyapatite paste, which is then sanded down until flush with the native bone. The temporalis muscle is resuspended along the superior temporal line. Due to dissection of the periosteum off the superior and lateral orbital rim, the orbicularis oculi muscle and superficial tissues tend to sag post-operatively, resulting in a partial ptosis with a cosmetic impact and occasional obscuration of vision. To avoid this, a bone-anchored suture is attached to the orbicularis oculi near the lateral canthus and used to suspend the muscle, resulting in a lateral brow lift. The wound is then closed in multiple layers. We prefer closure with a 4–0 Monocryl subcuticular stitch in the skin, and a 5–0 Proline running suture over the top which is removed on post-operative day 7. Representative pre- and post-operative MRI images are displayed in Fig. 3.

The traditional (two-piece) FO performed in our series is described in detail elsewhere [28]. Briefly, we elevate a three-quarters coronal skin flap and perform an interfascial dissection to preserve the frontalis nerve. The temporalis is then elevated from the temporal fossa to allow exposure of the lateral orbital wall. Then we perform a frontotemporal craniotomy. The dimensions of this craniotomy can be tailored to the pathology. The dura and periorbital are dissected, and then the orbitozygomatic bone flap involving the orbital roof, lateral orbital wall, orbital rim (bound by the supraorbital neurovascular bundle medially), and root of zygoma is cut and elevated separately. The greater sphenoid wing is then drilled down as necessary. This approach grants generous exposure of the subfrontal corridor to the parasellar region, and access to a trans-sylvian approach.

## Results

### Case series

Ten patients with ACMs and TSMs were included in this case series. (Table 1) Six patients underwent modified cranio-orbital craniotomy as described. The remaining four underwent traditional FO. Visual impairment was present in 7 of 10 patients. Tumor diameter, involvement of neurovascular structures, and tumor grade were similar between the two groups.

**Table 1:** Patient characteristics and outcomes of surgery

Patient number	Age/sex	Approach	Tumor location	Maximum tumor diameter (cm)	Optic canal invasion	Major vessel encasement	Cavernous sinus involvement	Presenting symptoms	Extent of resection	Site of residual disease	Complications	Visual outcome	Recurrence	Follow-up (months)
1	54/F	CO	TS	1.5	Y	N	N	Bilateral superior arcuate field cuts and decreased visual acuity	STR	Optic canal and ICA	-	→	-	7.9
2	63/M	CO	AC	2.6	N	ICA	Y	L inferior arcuate scotoma, memory deficit	STR	CS, Tentorial incisure	-	→	-	18
3	67/F	CO	TS	3	Y	ICA	Y	L monocular vision loss, L sided facial numbness, headaches	STR	CS, Tentorial incisure	-	↑	-	9
4	66/M	CO	AC/DS	2.3	N	N	Y	Incidental	STR	DS, CS	-	← →	-	43.2
5	55/F	CO	TS/PS	2.4	N	ICA	N	L monocular vision loss	GTR		R superior scotoma	↑	-	5.7
6	50/F	CO	AC	2.6	N	ICA	N	Incidental	STR	AC	-	← →	-	33.5
7	42/F	FO	AC	4.8	Y	ICA, MCA, ACA	N	R monocular vision loss and CN VI palsy	STR	ICA, MCA and ACA	Transient hyponatremia, numbness along upper lip in V2/V3	↑	-	52
8	69/F	FO	TS/PS	1.2	Y	N	Y	R monocular vision loss and progressive hearing loss	STR	CS, Optic Canal	-	↑	-	35
9	47/F	FO	TS	4	Y	N	Y	L monocular vision loss and mild proptosis, dysarthria, headaches	STR	CS, Meckel's cave	Facial numbness and V1/V2 trigeminal dysethesia	↑	-	6
10	52/F	FO	AC	4.7	N	ICA, MCA, ACA	Y	Seizure with preceding aura of dizziness and nausea	STR	CS, CN III & CN IV	V1 neuropathy, temporal wasting, mild L frontalis palsy, transient diplopia	← →	Slight interval increase	30
*P*-value				0.20	0.31	> 0.99	0.57		> 0.99		0.19	0.43	0.40	0.20

*AC* anterior clinoid, *ACA* anterior cerebral artery, *CO* modified cranio-orbital craniotomy, *CS* cavernous sinus, *DS* diaphragma sellae, *FO* traditional fronto-orbital craniotomy, *GTR* gross total resection, *ICA* internal carotid artery, *MCA* middle cerebral artery, *PS* planum sphenoidale, *STR* subtotal resection, *TS* tuberculum sellae; ↑ = impaired vision improved postoperatively; →  = impaired vision unchanged postoperatively; ←  →  = intact vision unchanged postoperatively

Of patients with impaired vision pre-op, 2/4 patients in the modified cranio-orbital group had improved vision and 2/4 had stable vision at last follow-up, compared to 3/3 with improved vision in the FO group (*p* = 0.429). There was no instance of postoperative visual deterioration. Patient 5 (cranio-orbital) developed a new superior scotoma in the R eye following surgery, however, this patient had presented with near complete loss of L sided vision, which was significantly improved following surgery. Overall, the patient endorsed improved vision postoperatively. Patient 1 (cranio-orbital) presented with recurrent tumor, which had been treated with surgery 20 years prior and fractionated radiotherapy 10 years prior. She was lost to follow-up and re-presented with over a year of progressive bilateral vision loss. Patient 2 (cranio-orbital) had a medical history of type II diabetes and hypertension and presented with several months of vision abnormalities in his left eye. Pre-operative exam established left inferior peripheral field cut with intact acuity bilaterally. His ophthalmologist concluded that ischemic optic neuropathy contributed to his stable postoperative visual deficits.

GTR was achieved in one (17%) case operated on with the modified cranio-orbital approach and in no cases in the FO group (*p* > 0.999). The most common site of residual disease was the cavernous sinus in 6 patients, 3 in each group.

Complications were mild and occurred in 16.7% of modified cranio-orbital patients and 75% of FO patients (*p* = 0.191). Complications in the modified cranio-orbital group were isolated to the R superior scotoma in patient 5 discussed above. Three FO patients had complications related to the superficial tissue dissection, including trigeminal dysesthesia, partial facial numbness, and a frontalis palsy. In contrast, no patient in the modified cranio-orbital group had a complication related to superficial tissue dissection (*p* = 0.033). There were no cases of tumor recurrence requiring re-intervention (*p* > 0.999).

### Literature review

The studies included in the literature review tabulated in Supplementary Table S1. In the TC group, three studies involved prospective data [17, 32, 38]. The remaining studies were retrospective. The most common TC approaches and presenting symptoms are summarized in Tables 2 and 3, respectively. (Table 4) Patients in the EEA group were significantly less likely to have optic canal invasion compared to TC or SoK patients (TC: 47.9%; EEA: 29.5%; SoK: 39.3%; TC vs. EEA *p* < 0.001; TC vs. SoK *p* = 0.082; EEA vs. SoK *p* = 0.040), and patients in the SoK group were significantly more likely to have major vessel encasement than TC and EEA patients (TC: 37.7%; EEA: 41.5%; SoK: 66.2%; TC vs. EEA *p* = 0.313; TC vs. SoK *p* < 0.001; EEA vs. SoK *p* < 0.001). Visual impairment at presentation was significantly more common in TC patients compared to EEA patients and SoK patients and in EEA compared to SoK patients (TC: 83.4%; EEA: 79.5%; SoK: 72.1%; TC vs. EEA *p* = 0.010; TC vs. SoK *p* < 0.001; EEA vs. SoK *p* < 0.001). Mean tumor diameter tended to be lower in EEA patients compared to the other groups (TC: 2.94 cm; EEA: 2.25 cm; SoK: 2.79 cm).

**Table 2:** Transcranial approaches for ACMs and TSMs in the literature

Approach	Number of studies	Patients operated on with this approach, N (%)
Pterional/frontotemporal	26	1157 (49.8)
Unilateral subfrontal	22	743 (32.0)
Unilateral subfrontal	15	380 (16.4)
Frontolateral	6	183 (7.9)
Lateral supraorbital	4	180 (7.7)
Orbitozygomatic/frontoorbitozygomatic	8	109 (4.7)
Frontoorbital, cranioorbital, supraorbital	5	113 (4.9)
Bifrontal	10	107 (4.6)
Interhemispheric	9	95 (4.1)

**Table 3:** Most common presenting symptoms in the literature

Symptom	N/N Total	%
Visual impairment	2810/3427	82.0
Headache	578/2420	23.9
Cognitive disturbance	76/2420	3.1
Seizure	90/2420	3.7
Hypothalamic pituitary axis impairment	36/2420	1.5
Limb weakness or hemiparesis	30/2420	1.2
Anosmia/hyposmia	13/2420	0.5
Dizziness	44/2420	1.8
Diplopia or extraocular cranial nerve deficit	26/2420	1.1
Other cranial nerve injury	60/2420	3.1

**Table 4:** Tumor characteristics from literature review

	All Studies		Transcranial		Endoscopic Endonasal Approach		Supraorbital Keyhole		*P*-value	Post hoc comparison P-value		
	N/N total	%	N/N total	%	N/N total	%	N/N total	%		TC vs. EEA	TC vs. SoK	EEA vs. SoK
Optic canal invasion	1118/2636	42.4	860/1796	47.9	216/733	29.5	42/107	39.3	** < 0.001**	** < 0.001**	0.082	**0.040**
Major vessel encasement	496/1245	39.8	366/971	37.7	83/200	41.5	47/74	66.2	** < 0.001**	0.313	** < 0.001**	**0.001**
Cavernous sinus involvement	189/631	30.0	176/583	30.2	9/32	28.1	4/16	25.0	0.8810	-	-	-
Visual impairment at presentation	2810/3427	82	2032/2435	83.4	672/845	79.5	106/147	72.1	** < 0.001**	**0.010**	** < 0.001**	**0.010**
Mean diameter (cm)	2.73		2.94		2.25		2.79		-	-	-	-

In patients with impaired vision preoperatively, the EEA group had significantly higher rates of visual improvement (Table 5). Vision improved at a rate of 57.8% in TC, 81.4% in EEA, and 68.2% in SoK patients (TC vs. EEA *p* < 0.001; TC vs. SoK *p* = 0.034; EEA vs. SoK *p* = 0.002). Rates of visual worsening and gross total resection were similar across all groups. Similar results were obtained when patients with intact pre-operative vision were included.

**Table 5:** Outcomes and complications from literature review

	Transcranial		Endoscopic Endonasal Approach		Supraorbital Keyhole		*P*-value	Post hoc comparison P-value		
	N/N total	%	N/N total	%	N/N total	%		TC vs. EEA	TC vs. SoK	EEA vs. SoK
Patients with visual deficit							** < 0.001**	-	-	-
Improved vision	883/1393	57.8	437/598	81.4	73/107	68.2	-	** < 0.001**	**0.034**	**0.002**
Stable vision	500/1393	32.7	72/598	13.4	26/107	24.3	-	** < 0.001**	0.071	**0.004**
Worsened vision	145/1393	9.5	28/598	5.2	8/107	7.5	-	**0.002**	0.490	0.352
All patients							** < 0.001**			
Improved vision	886/1794	49.4	522/763	68.4	73/140	52.1	-	** < 0.001**	0.530	** < 0.001**
Stable vision	723/1794	40.3	204/763	26.7	58/140	41.4	-	** < 0.001**	0.793	** < 0.001**
Worsened vision	185/1794	10.3	37/763	4.8	9/140	6.4	-	** < 0.001**	0.141	0.435
Gross total resection	1797/2367	75.9	647/844	76.7	118/147	80.3	0.465	-	-	-
Death	19/2157	0.9	2/786	0.2	1/147	0.7	0.202	-	-	-
CSF Leak	53/2157	2.5	134/786	17.0	3/147	2.0	** < 0.001**	** < 0.001**	0.751	** < 0.001**
Meningitis/Infection	47/2157	2.2	23/786	2.9	2/147	1.4	0.359	-	-	-
Stroke/new neurological deficit	69/2157	3.2	9/786	1.1	4/147	2.7	**0.009**	**0.002**	0.749	0.135
Vascular injury/hematoma	40/2157	1.9	7/786	0.9	2/147	1.4	0.119	-	-	-
Endocrinological complication	126/2157	5.8	22/786	2.8	3/147	2	** < 0.001**	** < 0.001**	0.053	0.601
Seizure	26/2157	1.2	4/786	0.5	0/147	0	0.110	-	-	-
Tumor recurrence	144/1911	7.5	58/701	8.2	4/109	2.7	0.238	-	-	-

The most frequently reported complication in any group was CSF leak in the EEA group. CSF leaks were significantly more common in EEA patients than in TC and SoK patients, with rates of 17.0%, 2.5%, and 2.0%, respectively (Table 5, TC vs. EEA *p* < 0.001; TC vs. SoK *p* = 0.751; EEA vs. SoK *p* < 0.001). Stroke or new neurological deficit occurred less frequently in EEA (1.1%) than TC (3.2%) patients, but was otherwise similar between groups (TC vs. EEA *p* = 0.002). Endocrinological complications were more common in TC (5.8%) patients compared to EEA (2.8%) patients (TC vs. EEA *p* < 0.001). There was no significant difference between groups in infectious complications, vascular injury/hematoma, seizures, mortality, or tumor recurrence.

There was no significant difference in tumor characteristics or vision outcomes amongst patients with visual deficits between our modified cranio-orbital group and any of the literature review groups (Table 6). Our modified cranio-orbital group had significantly lower rates of GTR than all three of the literature review groups, but recurrence rates were not significantly different.

**Table 6:** Comparison of modified cranio-orbital craniotomy vs. approaches from literature review

	All Studies		Transcranial		Endoscopic Endonasal Approach		Keyhole	
	OR	*P*-value	OR	*P*-value	OR	*P*-value	OR	*P*-value
Optic canal invasion	0.679	0.653	0.544	0.476	1.197	0.836	0.774	0.772
Major vessel encasement	3.020	0.181	3.306	0.145	2.723	0.219	1.149	0.877
Cavernous sinus involvement	2.339	0.287	2.313	0.294	2.556	0.290	3.000	0.262
Patients with visual deficit	0.439	0.329	0.397	0.270	0.515	0.437	0.774	0.771
Visual Outcome	-	-	-	0.675	-	0.101	-	0.473
Gross total resection	-	-	**0.063**	** < 0.001**	**0.061**	** < 0.001**	**0.049**	** < 0.001**
Tumor recurrence	-	-	0.0	0.534	0.0	0.462	0.0	0.633

## Discussion

We describe a minimally invasive modified cranio-orbital craniotomy through an eyebrow incision and demonstrate its safety and efficacy for resection of ACMs and TSMs. In our experience, selected ACMs and TSMs can be optimally approached with an eyebrow incision and an orbitofrontal bone flap that extends from the lateral margin of the supraorbital notch to the lateral orbital rim and includes the orbital roof with piecemeal removal extending to the ACP. Extradural clinoidectomy is generally performed to remove hyperostotic bone that serves as a vascular supply to the tumor and may contribute to recurrence. Clinoidectomy also opens the lateral wall of the optic canal to reduce tension on the optic nerve during subsequent manipulation. Whether or not it is seen on MRI, small amounts of tumor confined within a closed optic canal can impair blood flow and lead to visual deterioration. Bony decompression of the optic canal is the critical maneuver for preservation and improvement in vision.

Removal of the orbital roof to the level of the ACP during the approach allows for visualization of the optic nerve sheath entering and leaving the optic canal, which minimizes the risk to the optic nerve while the ACP and roof of the canal are drilled. ACMs and TSMs originate from the dura beneath the optic apparatus and vessels of the circle of Willis, displacing these structures superiorly. The inferior-to-superior trajectory afforded by removal of the orbital rim and roof allows for excellent access to the superior interface of the tumor with the neurovascular structures of interest, without significant brain retraction, and increases the available operative angles compared to the standard SoK approach. The subfrontal corridor to the tumor granted by this approach is limited by the orbital contents inferiorly, the supraorbital neurovascular bundle medially, the frontal lobe superiorly, and the lateral margin of the McCarty bur hole laterally. The traditional FO craniotomy provides a similar subfrontal corridor, with the additional option of a lateral subfrontal or trans-sylvian approach to ACMs and TSMs. Therefore, the traditional FO craniotomy should be used instead of the modified cranio-orbital approach when lateral subfrontal or trans-sylvian access to the perimesencephalic, pre-pontine, or suprasellar cisterns is needed.

Variations of orbitofrontal craniotomies have been described in the literature, usually utilizing a bicoronal incision for treatment of vascular lesions [4–6, 10, 11, 29, 33]. These descriptions differ in the skin incision used and the margins of the craniotomy. One report by Shanno et al. describes their experience with a “transorbital roof craniotomy” similar to our approach via an eyebrow incision in 72 patients, including 9 TSMs and ACMs [29]. Their craniotomy extends from the supraorbital notch medially, to the frontozygomatic suture laterally, and involves a 3.5 × 2.5 cm orbitofrontal bone flap. The authors report a Simpson Grade I or II resection in 7 of the 9 ACM and TSM cases (77.8%), with improved vision in 2 of 6 cases (33.3%). Overall, CSF rhinorrhea was the most common complication, in 6.9% of patients, but the only complication in ACM and TSM cases was one corneal abrasion. No other case series from our literature review described a minimally invasive orbitofrontal craniotomy through an eyebrow incision with detailed outcomes for meningiomas.

Visual outcomes in our series of patients in the supraorbital group were excellent, with 2/4 patients who presented with visual deficits having postoperative improvement in vision, and no instances of postoperative visual deterioration. Both patients with preoperative deficits who did not improve postoperatively had concurrent factors that limited the potential for improvement. Patient 1 had over 1 year of visual deterioration from recurrent tumor that was originally resected 20 years prior and irradiated after recurrence 10 years prior. Duration of visual deficit of > 7 months has been associated with worse visual outcomes [27, 39]. Patient 2 had concomitant hypertension and diabetes which contributed to ischemic optic neuropathy. There was no significant difference in visual improvement rates compared to patients operated via the FO approach. The rate of visual improvement in patients with preoperative visual impairment in our modified cranio-orbital patients was lower, but not significantly different from the rates for the TC, EEA, and SoK groups from the literature. The lower rate of improvement reported in our series may be related to small sample size and the comorbidities discussed above.

In our literature review, the rate of visual improvement for EEA patients was higher than those for TC or SoK. Published case series that directly compared TC to EEA approaches also found that EEA has either equivalent or better visual outcomes [13–15, 19, 21, 30]. Advantages of EEA include direct access to the tumor and its blood supply without manipulation of overlying vessels or brain retraction, and access to medial and inferior walls of the optic canal allowing decompression away from the region of tumor infiltration [13, 30]. However, EEA approaches are limited in their ability to resect tumors with vascular encasement or extension lateral to the ICA. These limitations are reflected in the smaller tumor diameters and the lower rates of vascular encasement in EEA tumors. Larger tumors have been associated with worse visual outcomes following tumor resection regardless of approach [12, 15]. Selection of smaller tumors for resection via EEA likely plays a role in the higher rate of reported visual improvement. The benefit of our modified cranio-oribital keyhole craniotomy with anterior clinoidectomy is it accomplishes the same degree of optic canal decompression while minimizing tissue disruption and allows greater direct control of tumor dissection off vessels at the skull base.

The GTR rate of 16.7% for our modified cranio-orbital patients is less than the GTR rates for the TC, EEA, and SoK groups from the literature review, which do not differ significantly from each other. We attribute the lower rate of GTR in our study to the characteristics of the tumors in our cohort, and our conservative definition of GTR. In this study, we defined GTR as a Simpson grade I or II resection, indicating resection of all gross tumor and resection or coagulation of all involved dura. In our cohort, optic canal invasion was present in 50% of patients and cavernous sinus invasion was encountered in 60%. After resection of all bulky tumor, the optic nerve sheath was not removed or coagulated, nor were the internal contents of the cavernous sinus to avoid any post-operative cranial nerve dysfunction. Therefore, even when post-operative MRI did not clearly identify residual disease, resections were deemed subtotal if residual dural involvement was seen at the time of surgery. With the availability of effective postoperative adjuvant or salvage radiotherapy, [3, 7, 9, 36] and recent evidence that suggests extent of resection has a smaller impact on tumor recurrence than previously thought [22, 34], we advocate for our conservative approach that does not risk new neurological deficit in order to resect small amounts of residual tumor.

Impaired ability to achieve complete resection due to limited maneuverability in the traditional SoK approach has been a concern in the adoption of minimally invasive surgery. Our review of the literature suggests that when appropriately applied, minimally invasive approaches can achieve similar extent of resection compared to TC or EEA approaches. The modified cranio-orbital approach is optimal for benign lesions with effective salvage treatment options for recurrence, such as low-grade meningiomas. Because the modified cranio-orbital approach does not afford the same lateral and trans-sylvian access as the traditional FO, it should not be used for malignant pathologies where extent of resection must be maximized.

Complication rates were low in our series, with no significant overall difference between the modified cranio-orbital group and the FO group. The FO group had postoperative complications related to dissection of superficial tissue more frequently than the modified cranio-orbital group based on this small, unstratified sample. This difference suggests that the decreased soft tissue manipulation afforded by the modified cranio-orbital approach with an eyebrow incision may be effective in reducing damage to superficial nerves.

In the literature review, CSF leak was encountered a higher rate in EEA patients. This finding is consistent with those in prior case series that include both EEA and TC patients [13–15, 30]. EEA patients from the literature review were less likely to experience postoperative stroke or new neurological deficit. This may be related to an inherent advantage of the EEA approach, or it may be caused by selection for meningiomas without complex vascular involvement.

Limitations of this study include the small size of the case series which limited the power of statistical comparisons and the retrospective design of the case series and literature review. Follow-up times in our case series were short in some cases, and longer follow-up times are needed in future studies to better evaluate tumor recurrence. Therefore, our results should be considered exploratory in nature, and further study is needed to delineate the advantages and disadvantages of this minimally invasive approach. Additional modifications to our approach, including a contralateral approach to minimize manipulation of the optic nerve, and use of the endoscope to assess for residual tumor out of sight of the microscope could be considered in future studies. In our analysis of the literature, complications and presenting symptoms were recorded differently for different studies, and patient characteristics could not be matched between groups. Our results are influenced by selection bias, as the anatomical characteristics of the cases reported in the literature affect the surgeon’s choice of approach. Specifically, tumors that extend laterally to the internal carotid artery, including ACMs, are not typically considered candidates for EEA approach. This constrains the conclusions that can be drawn from the statistical comparisons between SoK, EEA, and TC groups in our literature review. Disaggregated data was not broadly available in the literature, which limited univariate analysis for continuous variables and prevented multivariate analysis. Nevertheless, the concurrent analysis of our series and the literature review provides evidence that the modified cranio-orbital approach described here is safe and effective for resection of ACMs and TSMs, and provides a useful comparison of the general advantages and drawbacks of each technique.

## Conclusion

Our described modified cranio-orbital approach is an infrequently reported approach to ACMs and TSMs which includes removal of the orbital rim and roof to improve visualization and maneuverability while minimizing tissue exposure and brain retraction. We reported our experience with this approach and found similar visual outcomes, resection rates, and a lower rate of complications related to superficial tissue dissection when compared to traditional FO craniotomy. A review of the literature demonstrated that SoK approaches have similar rates of GTR to TC and EEA approaches, similar visual outcomes to TC approaches, and lower rates of postoperative CSF leak when compared to EEA approaches. The minimally invasive modified cranio-orbital approach through an eyebrow incision is a safe and effective approach that should be considered for resection of ACMs and TSMs.

## Supplementary Information

Below is the link to the electronic supplementary material.

ESM 1 Supplementary Material 1 (DOCX 126 KB)

## Data Availability

The data used in this article was generated through retrospective review of the electronic medical record and systematic review of the literature. Please contact the corresponding author for questions regarding this data.

## Abbreviations

ACM: Anterior clinoid meningioma
ACP: Anterior clinoid process
EEA: Endoscopic endonasal approach
FO: Fronto-orbital
GTR: Gross total resection
IQR: Interquartile range
OR: Odds ratio
OZ: Orbitozygomatic
SoK: Supraorbital keyhole
TC: Transcranial
MRI: Magnetic resonance imaging

## References

[1] Burks JD, Conner AK, Bonney PA et al (2016) Management of intracranial meningiomas using keyhole techniques. Cureus 8(4):e588. 10.7759/cureus.588 PMC4889455 27284496

[2] Cai M, Hou B, Luo L, Zhang B, Guo Y (2019) Trans-eyebrow supraorbital keyhole approach to tuberculum sellae meningiomas: a series of 30 cases with long-term visual outcomes and recurrence rates. J Neurooncol 142(3):545–555. 10.1007/s11060-019-03128-9 30796744

[3] Cohen-Inbar O, Lee CC, Schlesinger D, Xu Z, Sheehan JP (2016) Long-term results of stereotactic radiosurgery for skull base meningiomas. Neurosurgery 79(1):58–68. 10.1227/neu.0000000000001045 26421592

[4] Dare AO, Landi MK, Lopes DK, Grand W (2001) Eyebrow incision for combined orbital osteotomy and supraorbital minicraniotomy: application to aneurysms of the anterior circulation. Technical note. J Neurosurg 95(4):714–718. 10.3171/jns.2001.95.4.0714 11596969

[5] Delashaw JB Jr., Jane JA, Kassell NF, Luce C (1993) Supraorbital craniotomy by fracture of the anterior orbital roof. Technical note. J Neurosurg 79(4):615–8. 10.3171/jns.1993.79.4.0615 8410236

[6] Delfini R, Raco A, Artico M, Salvati M, Ciappetta P (1992) A two-step supraorbital approach to lesions of the orbital apex. Technical note. J Neurosurg 77(6):959–61. 10.3171/jns.1992.77.6.0959 1432142

[7] Di Franco R, Borzillo V, Ravo V et al (2018) Radiosurgery and stereotactic radiotherapy with cyberknife system for meningioma treatment. Neuroradiol J 31(1):18–26. 10.1177/1971400917744885 PMC5790000 29206077

[8] Fatemi N, Dusick JR, de Paiva Neto MA, Malkasian D, Kelly DF (2009) Endonasal versus supraorbital keyhole removal of craniopharyngiomas and tuberculum sellae meningiomas. Neurosurgery 64(5 Suppl 2):269–84; discussion 284–6. 10.1227/01.Neu.0000327857.22221.53 19287324

[9] Goldsmith BJ, Wara WM, Wilson CB, Larson DA (1994) Postoperative irradiation for subtotally resected meningiomas. A retrospective analysis of 140 patients treated from 1967 to 1990. J Neurosurg 80(2):195–201. 10.3171/jns.1994.80.2.0195 8283256

[10] Jane JA, Park EEA, Pobereskin LH, Winn HR, Butler AB (Oct1982) The supraorbital approach: technical note. Neurosurgery 11(4):537–542 7145070

[11] Jho HD (1997) Orbital roof craniotomy via an eyebrow incision: a simplified anterior skull base approach. Minim Invasive Neurosurg MIN 40(3):91–97. 10.1055/s-2008-1053424 9359086

[12] Karsy M, Raheja A, Eli I, Guan J, Couldwell WT (2017) Clinical outcomes with transcranial resection of the tuberculum sellae meningioma. World Neurosurg 108:748–755. 10.1016/j.wneu.2017.09.090 28951179

[13] Linsler S, Fischer G, Skliarenko V, Stadie A, Oertel J (2017) Endoscopic assisted supraorbital keyhole approach or endoscopic endonasal approach in cases of tuberculum sellae meningioma: which surgical route should be favored? World Neurosurg 104:601–611. 10.1016/j.wneu.2017.05.023 28512043

[14] Magill ST, Schwartz TH, Couldwell WT et al (2023) International tuberculum sellae meningioma study: surgical outcomes and management trends. Neurosurgery 93(6):1259–1270. 10.1227/neu.0000000000002569 PMC12245236 37389475

[15] Magill ST, Morshed RA, Lucas CG et al (2018) Tuberculum sellae meningiomas: grading scale to assess surgical outcomes using the transcranial versus transsphenoidal approach. Neurosurg Focus 44(4):E9. 10.3171/2018.1.Focus17753 29606045

[16] Marx S, Clemens S, Schroeder HWS (2018) The value of endoscope assistance during transcranial surgery for tuberculum sellae meningiomas. J Neurosurg 128(1):32–39. 10.3171/2016.11.Jns16713 28387626

[17] Mathiesen T, Kihlstrom L (2006) Visual outcome of tuberculum sellae meningiomas after extradural optic nerve decompression. Neurosurgery 59(3):570–6; discussion 570–6. 10.1227/01.Neu.0000228683.79123.F9 16955039

[18] McLaughlin N, Ditzel Filho LF, Shahlaie K, Solari D, Kassam AB, Kelly DF (Aug2011) The supraorbital approach for recurrent or residual suprasellar tumors. Minimally invasive neurosurgery : MIN 54(4):155–161. 10.1055/s-0031-1284401 21922443

[19] Mortazavi MM, Brito da Silva H, Ferreira M Jr., Barber JK, Pridgeon JS, Sekhar LN (2016) Planum sphenoidale and tuberculum sellae meningiomas: operative nuances of a modern surgical technique with outcome and proposal of a new classification system. World Neurosurg 86:270–86. 10.1016/j.wneu.2015.09.043 26409085

[20] Ormond DR, Hadjipanayis CG (2013) The supraorbital keyhole craniotomy through an eyebrow incision: its origins and evolution. Minim Invasive Surg. 10.1155/2013/296469 PMC3723243 23936644

[21] Ottenhausen M, Rumalla K, Alalade AF et al (2018) Decision-making algorithm for minimally invasive approaches to anterior skull base meningiomas. Neurosurg Focus 44(4):E7. 10.3171/2018.1.Focus17734 29606040

[22] Oya S, Kawai K, Nakatomi H, Saito N (Jul2012) Significance of Simpson grading system in modern meningioma surgery: integration of the grade with MIB-1 labeling index as a key to predict the recurrence of WHO Grade I meningiomas. J Neurosurg 117(1):121–128. 10.3171/2012.3.Jns111945 22559847

[23] Pitskhelauri DI, Sanikidze AZ, Abramov IT et al (2017) [The trans-eyebrow supraorbital approach for removal of anterior cranial fossa and suprasellar meningiomas]. Vopr Neirokhir Im N N Burdenko 81(6):89–98. 10.17116/neiro201781689-98 29393291

[24] Raza SM, Effendi ST, DeMonte F (2014) Tuberculum sellae meningiomas: evolving surgical strategies. Curr Surg Rep 2(11):73. 10.1007/s40137-014-0073-y

[25] Reisch R, Perneczky A, Filippi R (2003) Surgical technique of the supraorbital key-hole craniotomy. Surg Neurol 59(3):223–227. 10.1016/s0090-3019(02)01037-6 12681560

[26] Reisch R, Perneczky A (2005) Ten-year experience with the supraorbital subfrontal approach through an eyebrow skin incision. Neurosurgery 57(4 Suppl):242–55; discussion 242–55. 10.1227/01.neu.0000178353.42777.2c 16234671

[27] Schick U, Hassler W (Jul2005) Surgical management of tuberculum sellae meningiomas: involvement of the optic canal and visual outcome. J Neurol Neurosurg Psychiatry 76(7):977–983. 10.1136/jnnp.2004.039974 PMC1739719 15965205

[28] Seçkin H, Avci E, Uluç K, Niemann D, Başkaya MK (2008) The work horse of skull base surgery: orbitozygomatic approach. Technique, modifications, and applications. Neurosurg Focus 25(6):E4. 10.3171/FOC.2008.25.12.E4 19035702

[29] Shanno G, Maus M, Bilyk J et al (2001) Image-guided transorbital roof craniotomy via a suprabrow approach: a surgical series of 72 patients. Neurosurgery 48(3):559–67; discussion 567–8. 10.1097/00006123-200103000-00020 11270546

[30] Song SW, Kim YH, Kim JW et al (2018) Outcomes after transcranial and endoscopic endonasal approach for tuberculum meningiomas-a retrospective comparison. World Neurosurg 109:e434–e445. 10.1016/j.wneu.2017.09.202 29017976

[31] Soni RS, Patel SK, Husain Q, Dahodwala MQ, Eloy JA, Liu JK (2014) From above or below: the controversy and historical evolution of tuberculum sellae meningioma resection from open to endoscopic skull base approaches. J Clin Neurosci Off J Neurosurg Soc Australas 21(4):559–568. 10.1016/j.jocn.2013.03.043 24231561

[32] Spektor S, Dotan S, Mizrahi CJ (2013) Safety of drilling for clinoidectomy and optic canal unroofing in anterior skull base surgery. Acta Neurochir 155(6):1017–1024. 10.1007/s00701-013-1704-2 23605256

[33] Steiger HJ, Schmid-Elsaesser R, Stummer W, Uhl E (2001) Transorbital keyhole approach to anterior communicating artery aneurysms. Neurosurgery 48(2):347–51; discussion 351–2. 10.1097/00006123-200102000-00021 11220378

[34] Sughrue ME, Kane AJ, Shangari G et al (Nov2010) The relevance of Simpson Grade I and II resection in modern neurosurgical treatment of World Health Organization Grade I meningiomas. J Neurosurg 113(5):1029–1035. 10.3171/2010.3.Jns091971 20380529

[35] Telera S, Carapella CM, Caroli F et al (2012) Supraorbital keyhole approach for removal of midline anterior cranial fossa meningiomas: a series of 20 consecutive cases. Neurosurg Rev 35(1):67–83; discussion 83. 10.1007/s10143-011-0340-7 21800054

[36] Wenkel E, Thornton AF, Finkelstein D et al (2000) Benign meningioma: partially resected, biopsied, and recurrent intracranial tumors treated with combined proton and photon radiotherapy. Int J Radiat Oncol Biol Phys 48(5):1363–70. 10.1016/s0360-3016(00)01411-5 11121635

[37] Xiao J, Wang W, Wang X et al (2018) Supraorbital keyhole approach to the sella and anterior skull base via a forehead wrinkle incision. World Neurosurg 109:e343–e351. 10.1016/j.wneu.2017.09.177 28989048

[38] Xu M, Xu J, Huang X, Chen D, Chen M, Zhong P (2019) Small extended bifrontal approach for midline anterior skull base meningiomas: our experience with 54 consecutive patients. World Neurosurg. 10.1016/j.wneu.2018.12.172 30639482

[39] Zevgaridis D, Medele RJ, Muller A, Hischa AC, Steiger HJ (2001) Meningiomas of the sellar region presenting with visual impairment: impact of various prognostic factors on surgical outcome in 62 patients. Acta Neurochir 143(5):471–476. 10.1007/s007010170076 11482697

