# Supplementary material for: Cranio-orbital craniotomy via an eyebrow incision for resection of anterior clinoid and tuberculum sella meningiomas
Source: Acta Neurochir (Wien). 2026 Aug 11;168(1):256. doi: 10.1007/s00701-026-06997-y (PMC13740031; doi:10.1007/s00701-026-06997-y)
Supplement: Supplementary file 1 [file 701_2026_6997_MOESM1_ESM.docx]

| **Supplementary Table 1.** Categorization of studies included in literature review | | |  |
| --- | --- | --- | --- |
| Transcranial studies | Patients | Supraorbital keyhole studies | Patients |
| Aftahy et al. Cancers (2020).^1^ | 17 | Burks et al. Cureus (2016).^2^ | 10 |
| Attia et al. J Neurosurg (2012).^3^ | 22 | Cai et al. J Neurooncol (2019).^4^ | 30 |
| Bassiouni et al. J Neurosurg (2009).^5^ | 106 | Dzhindzhikhadze et al. Asian J Neurosurg (2020).^6^ | 15 |
| Chen et al. World J Surg Oncol (2013).^7^ | 6 | Fatemi, et al. Neurosurg (2009).^8^ | 9 |
| Chokyu et al. J Neurosurg (2011).^9^ | 34 | Linsler et al. World Neurosurg (2017).^10^ | 16 |
| Cohen-Cohen et al. JNS (2024).^11^ | 52 | Marx et al. J Neurosurg (2018).^12^ | 8 |
| Curey et al. J Neurosurg (2012).^13^ | 20 | McLaughlin et al. Minim Invas Neurosurg (2011).^14^ | 7 |
| Czernicki et al. Neurol Neurochir (2015).^15^ | 30 | Pitskhelauri et al. Burdenko J Neurosurg (2017).^16^ | 7 |
| Duan et al. Acta Neurochiurgica (2024).^17^ | 208 | Sasaki et al. World Neurosurg (2023).^18^ | 6 |
| Engelhardt et al. World Neurosurg (2018).^19^ | 20 | Telera et al. Neurosurg Rev (2012).^20^ | 15 |
| Feng et al. Front Neurol (2023).^21^ | 39 | Xiao et al. World Neurosurg (2018).^22^ | 5 |
| Galal et al. Acta Neurochir (2010).^23^ | 21 | Zheng et al. Front Surg (2022).^24^ | 19 |
| Ganna et al. Br J Neurosurg (2009).^25^ | 24 |  |  |
| Giammattei et al. World Neurosurg (2019).^26^ | 18 | Total: 12 | Total: 147 |
| Grutza et al. J Neurosurg (2024).^27^ | 71 |  |  |
| Jang et al. Neurosurg Rev (2012).^28^ | 24 |  |  |
| Karsy et al. World Neurosurg (2017).^29^ | 49 | Endoscopic endonasal approach studies | Patients |
| Kim et al. J Neurosurg (2022).^30^ | 74 | Alam et al. Life (Basel) (2022).^31^ | 5 |
| Kong et al. J Neurosurg (2018).^32^ | 94 | Attia et al. Neurosurg (2012).^33^ | 4 |
| Kuga et al. World Neurosurg (2018).^34^ | 13 | Bove et al. J Neurosurg (2024).^35^ | 48 |
| Landeiro et al. Arq Neuropsiquiatr (2010).^36^ | 23 | Elshazly et al. Oper Neurosurg (2018).^37^ | 25 |
| Leclerc et al. Clin Neurol Neurosurg (2022).^38^ | 34 | Fatemi et al. Neurosurg (2009).^8^ | 14 |
| Lee et al. World Neurosurg (2016).^39^ | 100 | Feng et al. Front Neur (2023).^21^ | 45 |
| Lefevre et al. Operative Neurosurg (2020).^40^ | 112 | Gadgil et al. J Neurol Surg B Skull Base (2013).^41^ | 5 |
| Leveque et al. Neurochir (2011).^42^ | 18 | Hayashi et al. World Neurosurg (2017). | 22 |
| Li et al. Front Surg (2022).^43^ | 31 | Kong et al. J Neurosurg (2018).^32^ | 84 |
| Li-Hua et al. Clin Neurol Neurosurg (2011).^44^ | 67 | Koutourousiou et al. J Neurosurg (2014).^45^ | 75 |
| Liu et al. Chin Med J (2015).^46^ | 21 | Kuga et al. World Neurosurg (2018).^34^ | 7 |
| Liu et al. Clin Neurol Neurosurg (2014).^47^ | 27 | Li et al. Front Surg (2022).^43^ | 7 |
| Mahmoud et al. Neurosurg (2010).^48^ | 58 | Linsler et al. World Neurosurg (2017).^10^ | 6 |
| Margalit et al. J Neurol Skull Base B (2013).^49^ | 51 | Magill et al. Neurosurg (2023).^50^ | 318 |
| Marian-Magana et al. World Neurosurg X (2024).^51^ | 16 | Marian-Magana et al. World Neurosurg X (2024).^51^ | 13 |
| Mariniello et al. Acta Neurochir (2012).^52^ | 90 | Mo et al. World Neurosurg (2024).^53^ | 26 |
| Marx et al. J Neurosurg (2018).^12^ | 7 | Mortazavi et al. World Neurosurg (2016).^54^ | 2 |
| Mo et al. World Neurosurg (2024).^53^ | 23 | Ogawa et al. Acta Neurochir (2012).^55^ | 19 |
| Mortazavi et al. World Neurosurg (2016).^54^ | 25 | Ottenhausen et al. Neurosurg Focus (2018).^56^ | 32 |
| Mortini et al. Clin Neurol Neurosurg (2012).^57^ | 37 | Padhye et al. Otolaryngol Head Neck Surg (2012).^58^ | 4 |
| Nanda et al. Neurosurg Focus (2013).^59^ | 30 | Song et al. World Neurosurg (2018).^60^ | 44 |
| Nanda et al. Clin Neurol Neurosurg (2016).^61^ | 36 | Wang et al. World Neurosurg (2010).^62^ | 12 |
| Ottenhausen et al. Neurosurg Focus (2018).^56^ | 6 | Yu et al. Sci Rep (2021).^63^ | 40 |
| Palani et al. J Neurosci Rural Pract (2012).^64^ | 41 | Total: 23 | Total: 857 |
| Romani et al. Neurosurg (2011).^65^ | 73 |  |  |
| Romani et al. Neurosurg (2012).^66^ | 52 |  |  |
| Sade et al. Surg Neurol (2010).^67^ | 31 |  |  |
| Salgado Lopez et al. Neurocir (2017).^68^ | 53 |  |  |
| Seol et al. Acta Neurochirurgica (2013).^69^ | 86 |  |  |
| Song et al. World Neurosurg (2018).^60^ | 40 |  |  |
| Sughrue et al. Cureus (2015).^70^ | 29 |  |  |
| Terasaka et al. Neurosurg (2011).^71^ | 9 |  |  |
| Verma et al. Neurol India (2016).^72^ | 78 |  |  |
| Voznyak et al. Neurosurg Rev (2021).^73^ | 26 |  |  |
| Wilk et al. Turk Neurosurg (2016).^74^ | 18 |  |  |
| Wong et al. World Neurosurg (2021).^75^ | 5 |  |  |
| Xiao et al. Front Oncol (2021).^76^ | 10 |  |  |
| Xu et al. World Neurosurg (2019).^77^ | 25 |  |  |
| Zhou et al. Turk Neurosurg (2016).^78^ | 56 |  |  |
| Total: 56 | Total: 2386 |  |  |

1.	Aftahy AK, Barz M, Krauss P, et al. Midline Meningiomas of the Anterior Skull Base: Surgical Outcomes and a Decision-Making Algorithm for Classic Skull Base Approaches. *Cancers (Basel)*. Nov 3 2020;12(11)doi:10.3390/cancers12113243

2.	Burks JD, Conner AK, Bonney PA, et al. Management of Intracranial Meningiomas Using Keyhole Techniques. *Cureus*. Apr 27 2016;8(4):e588. doi:10.7759/cureus.588

3.	Attia M, Umansky F, Paldor I, Dotan S, Shoshan Y, Spektor S. Giant anterior clinoidal meningiomas: surgical technique and outcomes. *Journal of neurosurgery*. Oct 2012;117(4):654-65. doi:10.3171/2012.7.Jns111675

4.	Cai M, Hou B, Luo L, Zhang B, Guo Y. Trans-eyebrow supraorbital keyhole approach to tuberculum sellae meningiomas: a series of 30 cases with long-term visual outcomes and recurrence rates. *Journal of neuro-oncology*. May 2019;142(3):545-555. doi:10.1007/s11060-019-03128-9

5.	Bassiouni H, Asgari S, Sandalcioglu IE, Seifert V, Stolke D, Marquardt G. Anterior clinoidal meningiomas: functional outcome after microsurgical resection in a consecutive series of 106 patients. Clinical article. *Journal of neurosurgery*. Nov 2009;111(5):1078-90. doi:10.3171/2009.3.17685

6.	Dzhindzhikhadze RS, Dreval ON, Lazarev VA, Polyakov AV, Kambiev RL, Salyamova EI. Transpalpebral Approach for Microsurgical Removal of Tuberculum Sellae Meningiomas. *Asian J Neurosurg*. Jan-Mar 2020;15(1):98-106. doi:10.4103/ajns.AJNS_186_19

7.	Chen G, Wang Z, Zhou D. Lateral supraorbital approach applied to sellar tumors in 23 consecutive patients: the Suzhou experience from China. *World journal of surgical oncology*. Feb 21 2013;11:41. doi:10.1186/1477-7819-11-41

8.	Fatemi N, Dusick JR, de Paiva Neto MA, Malkasian D, Kelly DF. Endonasal versus supraorbital keyhole removal of craniopharyngiomas and tuberculum sellae meningiomas. *Neurosurgery*. May 2009;64(5 Suppl 2):269-84; discussion 284-6. doi:10.1227/01.Neu.0000327857.22221.53

9.	Chokyu I, Goto T, Ishibashi K, Nagata T, Ohata K. Bilateral subfrontal approach for tuberculum sellae meningiomas in long-term postoperative visual outcome. *Journal of neurosurgery*. Oct 2011;115(4):802-10. doi:10.3171/2011.5.Jns101812

10.	Linsler S, Fischer G, Skliarenko V, Stadie A, Oertel J. Endoscopic Assisted Supraorbital Keyhole Approach or Endoscopic Endonasal Approach in Cases of Tuberculum Sellae Meningioma: Which Surgical Route Should Be Favored? *World neurosurgery*. Aug 2017;104:601-611. doi:10.1016/j.wneu.2017.05.023

11.	Cohen-Cohen S, Graffeo CS, Botello-Hernandez E, et al. Anterior clinoid meningiomas: surgical results and proposed scoring system to predict visual outcomes. *J Neurosurg*. May 1 2024;140(5):1295-1304. doi:10.3171/2023.8.jns23894

12.	Marx S, Clemens S, Schroeder HWS. The value of endoscope assistance during transcranial surgery for tuberculum sellae meningiomas. *Journal of neurosurgery*. Jan 2018;128(1):32-39. doi:10.3171/2016.11.Jns16713

13.	Curey S, Derrey S, Hannequin P, et al. Validation of the superior interhemispheric approach for tuberculum sellae meningioma: clinical article. *Journal of neurosurgery*. Dec 2012;117(6):1013-21. doi:10.3171/2012.9.Jns12167

14.	McLaughlin N, Ditzel Filho LF, Shahlaie K, Solari D, Kassam AB, Kelly DF. The supraorbital approach for recurrent or residual suprasellar tumors. *Minimally invasive neurosurgery : MIN*. Aug 2011;54(4):155-61. doi:10.1055/s-0031-1284401

15.	Czernicki T, Kunert P, Nowak A, Marchel A. Results of surgical treatment of anterior clinoidal meningiomas - our experiences. *Neurologia i neurochirurgia polska*. 2015;49(1):29-35. doi:10.1016/j.pjnns.2015.01.003

16.	Pitskhelauri DI, Sanikidze AZ, Abramov IT, et al. [The trans-eyebrow supraorbital approach for removal of anterior cranial fossa and suprasellar meningiomas]. *Zhurnal voprosy neirokhirurgii imeni N N Burdenko*. 2017;81(6):89-98. Supraorbital'nyi chrezbrovnyi dostup dlia udaleniia meningiom perednei cherepnoi iamki i supraselliarnoi oblasti. doi:10.17116/neiro201781689-98

17.	Duan C, Song D, Wang F, et al. Factors influencing postoperative visual improvement in 208 patients with tuberculum sellae meningiomas. *Acta Neurochir (Wien)*. Mar 16 2024;166(1):140. doi:10.1007/s00701-024-06033-x

18.	Sasaki T, Morisako H, Ikegami M, Wardhana DW, Fernandez-Miranda JC, Goto T. Endoscopic Supraorbital Eyebrow Approach for Medium-Sized Tuberculum Sellae Meningiomas: A Cadaveric Stepwise Dissection, Technical Nuances, and Surgical Outcomes. *World Neurosurg*. Aug 2023;176:e40-e48. doi:10.1016/j.wneu.2023.03.063

19.	Engelhardt J, Namaki H, Mollier O, et al. Contralateral Transcranial Approach to Tuberculum Sellae Meningiomas: Long-Term Visual Outcomes and Recurrence Rates. *World neurosurgery*. Aug 2018;116:e1066-e1074. doi:10.1016/j.wneu.2018.05.166

20.	Telera S, Carapella CM, Caroli F, et al. Supraorbital keyhole approach for removal of midline anterior cranial fossa meningiomas: a series of 20 consecutive cases. *Neurosurgical review*. Jan 2012;35(1):67-83; discussion 83. doi:10.1007/s10143-011-0340-7

21.	Feng Z, Li C, Cao L, et al. Comparative analysis of outcomes following craniotomy and expanded endoscopic endonasal approach resection of tuberculum sellae meningiomas: a single-institution study. *Front Neurol*. 2023;14:1139968. doi:10.3389/fneur.2023.1139968

22.	Xiao J, Wang W, Wang X, et al. Supraorbital Keyhole Approach to the Sella and Anterior Skull Base via a Forehead Wrinkle Incision. *World neurosurgery*. Jan 2018;109:e343-e351. doi:10.1016/j.wneu.2017.09.177

23.	Galal A, Faisal A, Al-Werdany M, El Shehaby A, Lotfy T, Moharram H. Determinants of postoperative visual recovery in suprasellar meningiomas. *Acta neurochirurgica*. Jan 2010;152(1):69-77. doi:10.1007/s00701-009-0492-1

24.	Zheng X, Shao D, Li Y, et al. Keyhole supraorbital eyebrow approach for fully endoscopic resection of tuberculum sellae meningioma. *Front Surg*. 2022;9:971063. doi:10.3389/fsurg.2022.971063

25.	Ganna A, Dehdashti AR, Karabatsou K, Gentili F. Fronto-basal interhemispheric approach for tuberculum sellae meningiomas; long-term visual outcome. *British journal of neurosurgery*. Aug 2009;23(4):422-30. doi:10.1080/02688690902968836

26.	Giammattei L, Starnoni D, Levivier M, Messerer M, Daniel RT. Surgery for Clinoidal Meningiomas: Case Series and Meta-Analysis of Outcomes and Complications. *World neurosurgery*. Sep 2019;129:e700-e717. doi:10.1016/j.wneu.2019.05.253

27.	Grutza M, Dao Trong P, Zweckberger K, Unterberg A. Pterional approach for tuberculum sellae meningiomas: a 17-year single-center experience. *J Neurosurg*. Jun 1 2024;140(6):1576-1583. doi:10.3171/2023.9.jns231657

28.	Jang WY, Jung S, Jung TY, Moon KS, Kim IY. The contralateral subfrontal approach can simplify surgery and provide favorable visual outcome in tuberculum sellae meningiomas. *Neurosurgical review*. Oct 2012;35(4):601-7; discussion 607-8. doi:10.1007/s10143-012-0397-y

29.	Karsy M, Raheja A, Eli I, Guan J, Couldwell WT. Clinical Outcomes with Transcranial Resection of the Tuberculum Sellae Meningioma. *World neurosurgery*. Dec 2017;108:748-755. doi:10.1016/j.wneu.2017.09.090

30.	Kim YJ, Moon KS, Jang WY, Jung TY, Kim IY, Jung S. Contralateral subfrontal approach for tuberculum sellae meningioma: techniques and clinical outcomes. *J Neurosurg*. Mar 1 2023;138(3):598-609. doi:10.3171/2022.5.jns212899

31.	Alam S, Ferini G, Muhammad N, et al. Skull Base Approaches for Tuberculum Sellae Meningiomas: Institutional Experience in a Series of 34 Patients. *Life (Basel)*. Mar 28 2022;12(4)doi:10.3390/life12040492

32.	Kong DS, Hong CK, Hong SD, et al. Selection of endoscopic or transcranial surgery for tuberculum sellae meningiomas according to specific anatomical features: a retrospective multicenter analysis (KOSEN-002). *Journal of neurosurgery*. May 18 2018;130(3):838-847. doi:10.3171/2017.11.Jns171337

33.	Attia M, Kandasamy J, Jakimovski D, et al. The importance and timing of optic canal exploration and decompression during endoscopic endonasal resection of tuberculum sella and planum sphenoidale meningiomas. *Neurosurgery*. Sep 2012;71(1 Suppl Operative):58-67. doi:10.1227/NEU.0b013e318258e23d

34.	Kuga D, Toda M, Yoshida K. Treatment Strategy for Tuberculum Sellae Meningiomas Based on a Preoperative Radiological Assessment. *World Neurosurg*. Dec 2018;120:e1279-e1288. doi:10.1016/j.wneu.2018.09.054

35.	Bove I, Solari D, Colangelo M, et al. Analysis of visual impairment score in a series of 48 tuberculum sellae meningiomas operated on via the endoscopic endonasal approach. *J Neurosurg*. Mar 1 2024;140(3):696-704. doi:10.3171/2023.7.jns23437

36.	Landeiro JA, Goncalves MB, Guimaraes RD, et al. Tuberculum sellae meningiomas: surgical considerations. *Arquivos de neuro-psiquiatria*. Jun 2010;68(3):424-9.

37.	Elshazly K, Kshettry VR, Farrell CJ, Nyquist G, Rosen M, Evans JJ. Clinical Outcome after Endoscopic Endonasal Resection of Tuberculum Sella Meningiomas. *Oper Neurosurg (Hagerstown)*. May 1 2018;14(5):494-502. doi:10.1093/ons/opx165

38.	Leclerc A, Gaberel T, Laville MA, Derrey S, Quintyn JC, Emery E. Factors associated with favorable visual outcome after surgery of clinoidal meningiomas. *Clin Neurol Neurosurg*. Dec 2022;223:107508. doi:10.1016/j.clineuro.2022.107508

39.	Lee S, Hong SH, Cho YH, Kim JH, Kim CJ. Anatomical Origin of Tuberculum Sellae Meningioma: Off-Midline Location and Its Clinical Implications. *World neurosurgery*. May 2016;89:552-61. doi:10.1016/j.wneu.2016.02.016

40.	Lefevre E, Terrier LM, Bekaert O, et al. Microsurgical Transcranial Approach of 112 Paraoptic Meningiomas: A Single-Center Case Series. *Oper Neurosurg (Hagerstown)*. © 2020 by the Congress of Neurological Surgeons.; 2020:651-658. vol. 6.

41.	Gadgil N, Thomas JG, Takashima M, Yoshor D. Endoscopic resection of tuberculum sellae meningiomas. *Journal of neurological surgery Part B, Skull base*. Aug 2013;74(4):201-10. doi:10.1055/s-0033-1342922

42.	Leveque S, Derrey S, Martinaud O, et al. Superior interhemispheric approach for midline meningioma from the anterior cranial base. *Neuro-Chirurgie*. Jul 2011;57(3):105-13. doi:10.1016/j.neuchi.2011.08.001

43.	Li Y, Zhang C, Su J, Qin C, Wang X, Liu Q. Individualized surgical treatment of giant tuberculum sellae meningioma: Unilateral subfrontal approach vs. endoscopic transsphenoidal approach. *Front Surg*. 2022;9:990646. doi:10.3389/fsurg.2022.990646

44.	Li-Hua C, Ling C, Li-Xu L. Microsurgical management of tuberculum sellae meningiomas by the frontolateral approach: surgical technique and visual outcome. *Clinical neurology and neurosurgery*. Jan 2011;113(1):39-47. doi:10.1016/j.clineuro.2010.08.019

45.	Koutourousiou M, Fernandez-Miranda JC, Stefko ST, Wang EW, Snyderman CH, Gardner PA. Endoscopic endonasal surgery for suprasellar meningiomas: experience with 75 patients. *Journal of neurosurgery*. Jun 2014;120(6):1326-39. doi:10.3171/2014.2.Jns13767

46.	Liu HC, Qiu E, Zhang JL, et al. Surgical Indications of Exploring Optic Canal and Visual Prognostic Factors in Neurosurgical Treatment of Tuberculum Sellae Meningiomas. *Chinese medical journal*. Sep 5 2015;128(17):2307-11. doi:10.4103/0366-6999.163391

47.	Liu Y, Chotai S, Ming C, Jin S, Pan J, Qi S. Characteristics of midline suprasellar meningiomas based on their origin and growth pattern. *Clinical neurology and neurosurgery*. Oct 2014;125:173-81. doi:10.1016/j.clineuro.2014.08.002

48.	Mahmoud M, Nader R, Al-Mefty O. Optic canal involvement in tuberculum sellae meningiomas: influence on approach, recurrence, and visual recovery. *Neurosurgery*. Sep 2010;67(3 Suppl Operative):ons108-18; discussion ons118-9. doi:10.1227/01.Neu.0000383153.75695.24

49.	Margalit N, Shahar T, Barkay G, et al. Tuberculum sellae meningiomas: surgical technique, visual outcome, and prognostic factors in 51 cases. *J Neurol Surg B Skull Base*. Aug 2013;74(4):247-58. doi:10.1055/s-0033-1342920

50.	Magill ST, Schwartz TH, Couldwell WT, et al. International Tuberculum Sellae Meningioma Study: Surgical Outcomes and Management Trends. *Neurosurgery*. Dec 1 2023;93(6):1259-1270. doi:10.1227/neu.0000000000002569

51.	Marian-Magaña R, Sangrador-Deitos MV, Rodríguez-Hernández L, et al. Visual outcomes in tuberculum sellae meningiomas comparing transcranial and endoscopic endonasal approaches. *World Neurosurg X*. Jul 2024;23:100319. doi:10.1016/j.wnsx.2024.100319

52.	Mariniello G, de Divitiis O, Bonavolonta G, Maiuri F. Surgical unroofing of the optic canal and visual outcome in basal meningiomas. *Acta neurochirurgica*. Jan 2013;155(1):77-84. doi:10.1007/s00701-012-1485-z

53.	Mo J, Hasegawa H, Shin M, et al. Endoscopic Endonasal Approach Is Superior to Transcranial Approach for Small to Medium Tuberculum Sellae Meningiomas in Terms of Visual Outcome and Complications: A Retrospective Study in a Single Center. *World Neurosurg*. Sep 2024;189:e814-e824. doi:10.1016/j.wneu.2024.07.010

54.	Mortazavi MM, Brito da Silva H, Ferreira M, Jr., Barber JK, Pridgeon JS, Sekhar LN. Planum Sphenoidale and Tuberculum Sellae Meningiomas: Operative Nuances of a Modern Surgical Technique with Outcome and Proposal of a New Classification System. *World neurosurgery*. Feb 2016;86:270-86. doi:10.1016/j.wneu.2015.09.043

55.	Ogawa Y, Tominaga T. Extended transsphenoidal approach for tuberculum sellae meningioma--what are the optimum and critical indications? *Acta Neurochir (Wien)*. Apr 2012;154(4):621-6. doi:10.1007/s00701-011-1266-0

56.	Ottenhausen M, Rumalla K, Alalade AF, et al. Decision-making algorithm for minimally invasive approaches to anterior skull base meningiomas. *Neurosurgical focus*. Apr 2018;44(4):E7. doi:10.3171/2018.1.Focus17734

57.	Mortini P, Barzaghi LR, Serra C, Orlandi V, Bianchi S, Losa M. Visual outcome after fronto-temporo-orbito-zygomatic approach combined with early extradural and intradural optic nerve decompression in tuberculum and diaphragma sellae meningiomas. *Clinical neurology and neurosurgery*. Jul 2012;114(6):597-606. doi:10.1016/j.clineuro.2011.12.021

58.	Padhye V, Naidoo Y, Alexander H, et al. Endoscopic endonasal resection of anterior skull base meningiomas. *Otolaryngology--head and neck surgery : official journal of American Academy of Otolaryngology-Head and Neck Surgery*. Sep 2012;147(3):575-82. doi:10.1177/0194599812446565

59.	Nanda A, Ambekar S, Javalkar V, Sharma M. Technical nuances in the management of tuberculum sellae and diaphragma sellae meningiomas. *Neurosurgical focus*. Dec 2013;35(6):E7. doi:10.3171/2013.10.Focus13350

60.	Song SW, Kim YH, Kim JW, et al. Outcomes After Transcranial and Endoscopic Endonasal Approach for Tuberculum Meningiomas-A Retrospective Comparison. *World neurosurgery*. Jan 2018;109:e434-e445. doi:10.1016/j.wneu.2017.09.202

61.	Nanda A, Konar SK, Maiti TK, Bir SC, Guthikonda B. Stratification of predictive factors to assess resectability and surgical outcome in clinoidal meningioma. *Clinical neurology and neurosurgery*. Mar 2016;142:31-37. doi:10.1016/j.clineuro.2016.01.005

62.	Wang Q, Lu XJ, Ji WY, et al. Visual outcome after extended endoscopic endonasal transsphenoidal surgery for tuberculum sellae meningiomas. *World neurosurgery*. Jun 2010;73(6):694-700. doi:10.1016/j.wneu.2010.04.007

63.	Yu P, Xu T, Wu X, Liu Z, Wang Y. The expanded endoscopic endonasal approach for treatment of tuberculum sellae meningiomas in a series of 40 consecutive cases. *Sci Rep*. Mar 2 2021;11(1):4993. doi:10.1038/s41598-021-83905-7

64.	Palani A, Panigrahi MK, Purohit AK. Tuberculum sellae meningiomas: A series of 41 cases; surgical and ophthalmological outcomes with proposal of a new prognostic scoring system. *Journal of neurosciences in rural practice*. Sep 2012;3(3):286-93. doi:10.4103/0976-3147.102608

65.	Romani R, Laakso A, Kangasniemi M, Lehecka M, Hernesniemi J. Lateral supraorbital approach applied to anterior clinoidal meningiomas: experience with 73 consecutive patients. *Neurosurgery*. Jun 2011;68(6):1632-47; discussion 1647. doi:10.1227/NEU.0b013e318214a840

66.	Romani R, Laakso A, Kangasniemi M, Niemela M, Hernesniemi J. Lateral supraorbital approach applied to tuberculum sellae meningiomas: experience with 52 consecutive patients. *Neurosurgery*. Jun 2012;70(6):1504-18; discussion 1518-9. doi:10.1227/NEU.0b013e31824a36e8

67.	Sade B, Lee JH. High incidence of optic canal involvement in tuberculum sellae meningiomas: rationale for aggressive skull base approach. *Surgical neurology*. Aug 2009;72(2):118-23; discussion 123. doi:10.1016/j.surneu.2008.08.007

68.	Salgado Lopez L, Munoz Hernandez F, Asencio Cortes C, Tresserras Ribo P, Alvarez Holzapfel MJ, Molet Teixido J. Extradural anterior clinoidectomy in the management of parasellar meningiomas: Analysis of 13 years of experience and literature review. *Neurocirugia (Asturias, Spain)*. Sep - Oct 2018;29(5):225-232. Clinoidectomia anterior extradural en el manejo de meningiomas paraselares: analisis de 13 anos de experiencia y revision de la literatura. doi:10.1016/j.neucir.2018.04.002

69.	Seol HJ, Park HY, Nam DH, et al. Clinical outcomes of tuberculum sellae meningiomas focusing on reversibility of postoperative visual function. *Acta neurochirurgica*. Jan 2013;155(1):25-31. doi:10.1007/s00701-012-1551-6

70.	Sughrue M, Kane A, Rutkowski MJ, Berger MS, McDermott MW. Meningiomas of the Anterior Clinoid Process: Is It Wise to Drill Out the Optic Canal? *Cureus*. Sep 10 2015;7(9):e321. doi:10.7759/cureus.321

71.	Terasaka S, Asaoka K, Kobayashi H, Yamaguchi S. Anterior interhemispheric approach for tuberculum sellae meningioma. *Neurosurgery*. Mar 2011;68(1 Suppl Operative):84-8; discussion 88-9. doi:10.1227/NEU.0b013e31820781e1

72.	Verma SK, Sinha S, Sawarkar DP, et al. Medial sphenoid wing meningiomas: Experience with microsurgical resection over 5 years and a review of literature. *Neurology India*. May-Jun 2016;64(3):465-75. doi:10.4103/0028-3886.181548

73.	Voznyak O, Lytvynenko A, Maydannyk O, Ilyuk R, Zinkevych Y, Hryniv N. Tuberculum sellae meningioma surgery: visual outcomes and surgical aspects of contralateral approach. *Neurosurg Rev*. Apr 2021;44(2):995-1001. doi:10.1007/s10143-020-01278-3

74.	Wilk A, Zielinski G, Witek P, Koziarski A. Outcome Assessment After Surgical Treatment of Tuberculum Sellae Meningiomas- A Preliminary Report. *Turkish neurosurgery*. 2016;26(6):824-832. doi:10.5137/1019-5149.Jtn.14160-15.1

75.	Wong AK, Kramer DE, Wong RH. Keyhole Superior Interhemispheric Transfalcine Approach for Tuberculum Sellae Meningioma: Technical Nuances and Visual Outcomes. *World Neurosurg*. Jan 2021;145:5-12. doi:10.1016/j.wneu.2020.08.222

76.	Xiao F, Shen J, Zhang L, et al. Unilateral Subfrontal Approach for Giant Tuberculum Sellae Meningioma: Single Center Experience and Review of the Literature. *Front Oncol*. 2021;11:708235. doi:10.3389/fonc.2021.708235

77.	Xu M, Xu J, Huang X, Chen D, Chen M, Zhong P. Small Extended Bifrontal Approach for Midline Anterior Skull Base Meningiomas: Our Experience with 54 Consecutive Patients. *World neurosurgery*. Jan 11 2019;doi:10.1016/j.wneu.2018.12.172

78.	Zhou H, Wu Z, Wang L, Zhang J. Microsurgical Treatment of Tuberculum Sellae Meningiomas with Visual Impairments: A Chinese Experience of 56 Cases. *Turkish neurosurgery*. 2016;26(1):48-53. doi:10.5137/1019-5149.Jtn.11476-14.1
